# Enhancing Alkane Production in Cyanobacterial Lipid Droplets: A ModeFl Platform for Industrially Relevant Compound Production

**DOI:** 10.3390/life5021111

**Published:** 2015-03-26

**Authors:** Anantha Peramuna, Ray Morton, Michael L. Summers

**Affiliations:** Department of Biology, California State University Northridge, 18111 Nordhoff St., Northridge, CA 91330-8303, USA; E-Mails: anvp@plen.ku.dk (A.P.); rmort001@ucr.edu (R.M.)

**Keywords:** alkane, cyanobacteria, lipid droplet, acyl-acyl carrier protein reductase (Aar), aldehyde decarbonylase (Adc)

## Abstract

Cyanobacterial lipid droplets (LDs) are packed with hydrophobic energy-dense compounds and have great potential for biotechnological expression and the compartmentalization of high value compounds. *Nostoc punctiforme* normally accumulates LDs containing neutral lipids, and small amounts of heptadecane, during the stationary phase of growth. In this study, we further enhanced heptadecane production in *N. punctiforme* by introducing extrachromosomal copies of *aar/adc* genes, and report the discovery of a putative novel lipase encoded by Npun_F5141, which further enhanced alkane production. Extra copies of all three genes in high light conditions resulted in a 16-fold higher accumulation of heptadecane compared to the wild type strain in the exponential phase. LD accumulation during exponential phase also increased massively to accommodate the heptadecane production. A large number of small, less fluorescent LDs were observed at the cell periphery in exponential growth phase, whereas fewer number of highly fluorescent, much larger LDs were localized towards the center of the cell in the stationary phase. These advances demonstrate that cyanobacterial LDs are an ideal model platform to make industrially relevant compounds, such as alkanes, during exponential growth, and provide insight into LD formation in cyanobacteria.

## 1. Introduction

Production of biofuel and high-value compounds from the carbon dioxide of photosynthetic organisms has recently been of extreme interest [1,2,3,4]. Photosynthetic microbes have the capacity to out-produce plant-based production platforms, both in terms of the efficiency of solar conversion, and in land use [5,6]. Cyanobacteria are uniquely placed into this mix due to their relatively fast growth rate and wide range of available genetic tools. Filamentous cyanobacteria are of special interest due to the relative ease of harvest, relative to unicellular strains [6]. With the advent and use of metabolic engineering systems, it is becoming possible to design or modify organisms to synthesize a wide array of compounds. To harness this potential, it is becoming increasingly important to have a solid understanding of the metabolic trafficking and growth-state specific metabolic capabilities of these organisms so that the production of high-value compounds can be integrated into existing, or modified, metabolic pathways.

Lipid droplets (LDs) are proposed to have a neutral lipid core surrounded by a lipid mono-layer membrane containing polar or charged headgroups [7]. LDs in chloroplasts, known as plastoglobules (PGs), occur in all types of plant plastids, including chloroplasts [7]. In chloroplasts, PGs are mainly attached to thylakoid membranes, near their curved surfaces, with smaller numbers in the stroma [8,9]. The current model of formation invokes the budding or “blistering” of LDs from the outer leaf of the endoplasmic reticulum, or thylakoid membrane, due to the accumulation of neutral lipids between the leaflets of the phospholipid bilayer [10,11,12,13].

Recent work in our lab has identified ~300 nm diameter LDs in the vegetative cells of the cyanobacterium *Nostoc punctiforme*, which accumulate during the stationary phase and in the exponential phase, in the presence of exogenous carbohydrates. These can easily be purified from lysed cells by centrifugation due to their low density. Isolated LDs were enriched for alpha tochopherol, triacylglycerols (TAGs), containing saturated C16:0 and C18:0 fatty acyl groups, and an *n-*alkane containing 17 carbons (C17) [14]. LDs are an ideal discrete “packet” in which to deposit and accumulate hydrophobic compounds, and, due to their sequestration in the neutral lipid environment of the LD, could potentially avoid the toxic effects of these compounds.

Microalgae have the capability of producing triacylglycerides (TAGs) at high levels (commonly 20%–50% of dry weight) at rates of 10–200 times faster than those of terrestrial oil crops [15]. TAGs are the preferred feedstock for the production of biodiesel due to the absence of headgroups containing phosphorous or sulfur. Biodiesel, also known as fatty acid methyl esters (FAMEs), are produced from extracted triacylglycerols by transesterification, using methanol in the presence of a catalyst such as sodium hydroxide [16,17]. Currently, biodiesel is added to petroleum-based diesel at concentrations of up to 5% and can be used in any application as if it were pure petroleum diesel without requiring any modifications to equipment. Up to 20% can be added with minor modifications [18]. Biodiesels, containing saturated FAMEs, are desirable over unsaturated species due to their high cetane number (ignition quality) and high stability, yet they may be undesirable in cold conditions due to their higher viscosity. The production of alkanes from triacylglycerols is more difficult and involves pyrolysis and thermochemical liquefaction, with subsequent refinement to harvest the alkanes.

A value-added enhancement of photosynthetic biodiesel production from TAG would be the co-synthesis of alka(e)nes, the typical hydrocarbons found in petroleum-derived diesel. Due to their hydrophobicity, alka(e)nes co-purify with TAGs and are un-modified during the conversion to FAMEs. Alka(e)nes can be produced naturally by a diverse range of prokaryotes [19], including cyanobacteria [20,21]. Recently, two enzymes have been identified in cyanobacteria that can sequentially convert CO_2_-derived free fatty acids into alka(e)nes. The first enzyme, an acyl-acyl carrier protein reductase (Aar), reduces the fatty-acid-containing *n* carbons to a fatty ester. The second enzyme, an aldehyde decarbonylase (Adc), removes a carbon in the form of formate, and produces an alka(e)ne containing *n*−1 carbons. When these genes from cyanobacteria were expressed in *Escherichia coli*, a range of alka(e)nes, containing 13- to 17-carbons, were produced [22], which is within the range typical of biodiesels. Previous work in cyanobacteria has shown that the expression of two genomic copies of *aar* and *adc* in *Synechocystis* sp. PCC6803 resulted in an 8.3-fold increase in the production of alka(e)nes relative to wild-type levels [23].

Here we show that increasing the copy number of the native *aar* and *adc* genes results in large amounts of only one type of alkane, and this is deposited preferentially into lipid droplets of *N. punctiforme*. Alkane production can be further enhanced by combinations of high light and multiple copies of a novel gene encoding a putative lipase. Growth defects due to *aar* and *adc* can be reduced by media supplementation and novel gene expression, indicating that metabolic flux, and not alkane toxicity, is the limiting factor for higher alkane production in cyanobacteria.

## 2. Results

### 2.1. Multiple Copies of Aar/Adc Leads to Enhanced C17 Alkane Production Specifically in Lipid Droplets

**Figure 1:**
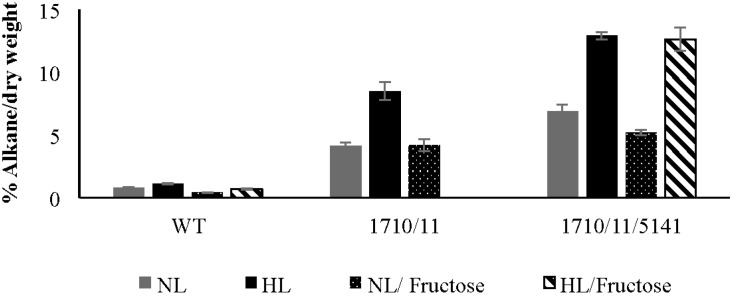
Effect of fructose and high light on total heptadecane production of wild-type (WT) and strains bearing multi-copy plasmids containing *aar/adc* (Npun_R1710/11) alone and in combination with Npun_F5141. Cultures were grown to exponential phase in media containing ammonia. Data expressed as a percent of C17 alkanes per cell dry weight (w/w), *n =* 3. NL, normal light; HL, high light; Fructose, indicates addition of 20 mM fructose to cultures.

**Figure 2:**
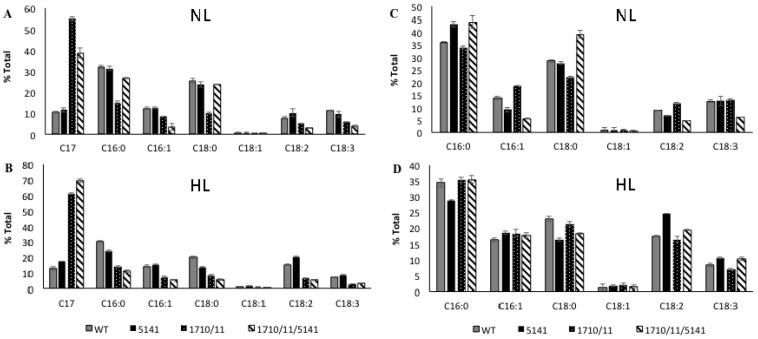
Relative expression of alkanes and FAMEs derived from neutral lipids extracted from exponential phase whole-cells under normal light (NL; (**A**) and (**C**)) and high light (HL: (**B**) and (**D**)) conditions. Wild-type strain (WT), and strains bearing plasmids expressing Npun_F5141 only, Npun_F1710/F1711, or all three genes. Data is plotted with ((**A**) and (**B**)) or without ((**C**) and (**D**)) inclusion of alkane data.

Strains of *N. punctiforme* containing or lacking extra copies of *aar/adc* genes encoded by Npun_F1710/Npun_F1711 expressed on a multicopy plasmid under control of their own promoter were compared. In standard growth medium supplemented with ammonia under normal laboratory light conditions (NL), the exponentially growing wild-type control strain accumulated 0.77% ± 0.06% heptadecane, a saturated *n-*alkane containing 17 carbons (C17) based on dry weight (Figure 1). C17 was the only alkane present in these cells; no other saturated or unsaturated alkanes were observed. The wild-type strain increased C17 production to 1.12% ± 0.06% of dry weight under high light (HL) conditions more typical of industrial settings. The Np1710/11 strain accumulated 4.11% ± 0.28% dry weight of C17 under normal growth conditions that increased to 8.48% ± 0.73% under high light conditions (Figure 1). The relative amount of C17 compared to FAMEs derived from whole cells was determined under exponential growth conditions, as above (Figure 2). Since absolute amounts of alkane production were already established (Figure 1), relative comparisons of FAME data were deemed sufficient to highlight the physiological changes due to overexpression of associated genes. In log phase NL the Np1710/11 strain contained 54.9% ± 0.73% heptadecane, whereas the wild-type only contained 10.4% ± 0.46% (Figure 2A). When C17 was left out of the analysis to observe the physiological effect on FAMEs arising from di- and triacylglycerol lipids, the relative amount of C18:0 was reduced significantly in the Np1710/11 strain under NL conditions (Figure 2C). Under HL conditions, all species of lipids appeared proportionally lower in the Np1710/11 strain relative to wild type due to the large proportion occurring as C17 alkanes (Figure 2C). When C17 was left out of the analysis, it was apparent that lipids were similar to that of the wild-type strain (Figure 2D).

**Figure 3:**
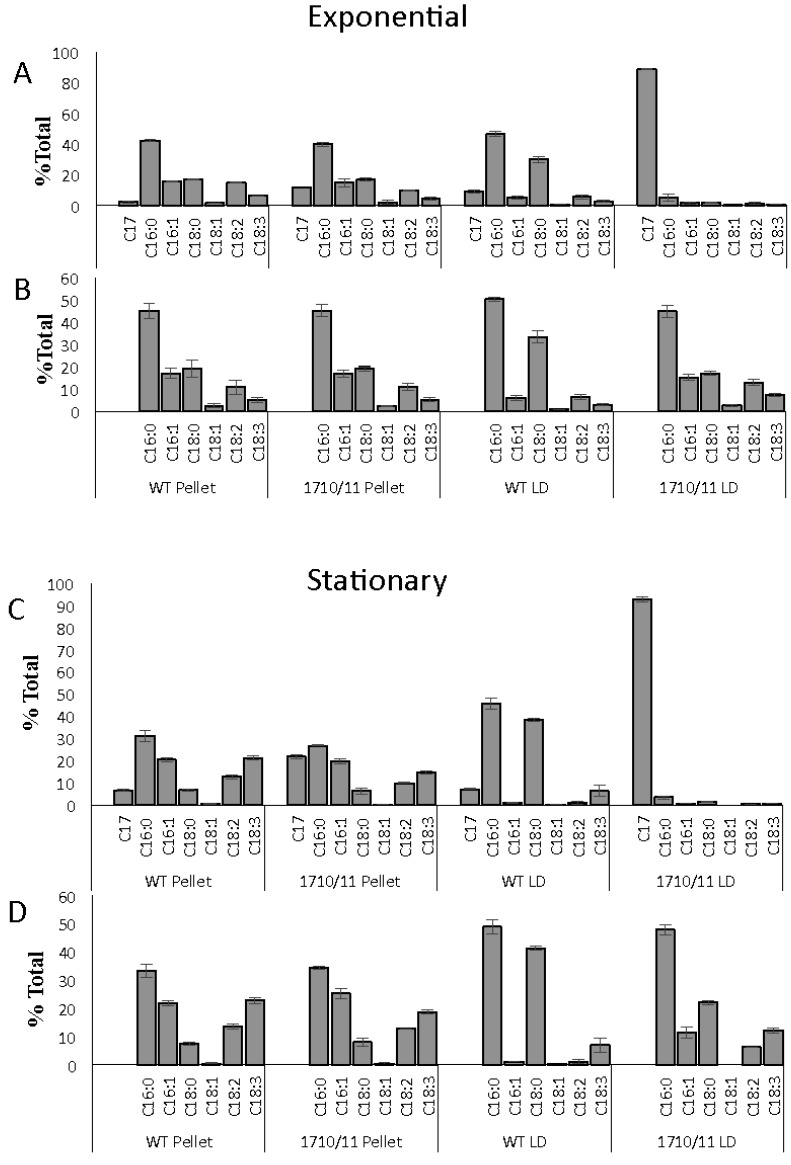
Relative amounts of heptadecane and FAMEs in lipids extracted from isolated lipid droplets and cell debris of wild-type and Npun_F1710/11 strains (*n =* 3) during exponential growth ((**A**) and (**B**)) and stationary phase ((**C**) and (**D**)). FAME composition with ((**A**) and (**C**)) or without ((**B**) and (**D**)) heptadecane data included.

To determine the cellular location of alkanes and their effects on membranes, lipids and alkanes, extracted from lipid droplets isolated by density centrifugation, were compared to lipids in the pellet containing cellular debris after LD removal. Both the exponential and stationary phase cultures were included in this analysis (Figure 3A,C). To more clearly visualize the effects of alkane over-production on fatty-acid-composition of membrane and LD lipids, the same data was plotted without heptadecane included in the analysis (Figure 3B,D). The data indicate that relative proportions of the various fatty acid species in lipids found in the pellet of the Np1710/11 strain did not differ from that of the wild-type when compared to each other during either the exponential or stationary phases (Figure 3B,D), although there was an increase in saturated fatty acids as cells entered stationary phase. C17 was present in pellet lipids of both wild-type and Np1710/11 strains during exponential phase (at 2.43% ± 0.60% and 12.0 ± 1.0, respectively; Figure 3C) with a higher proportion co-purifying with pellets from stationary phase cells (6.74% ± 0.60% and 21.8% ± 1.1%, respectively; Figure 3A), perhaps due to nascent membrane-associated LDs or C17 intercalated in membranes.

Comparisons among isolated LDs indicated C16:0 and C18:0 fatty acids were the predominant species in wild-type LDs, regardless of growth phase, representing 91% of total FAME-derived lipids from the exponential phase and 84% from the stationary phase (Figure 3B,D). LDs isolated from Np1710/11 had reduced amounts of C18:0 fatty acids relative to the wild-type strain, as expected if this species is drained due to formation of C17 alkanes (Figure 3B,D). C17 accounted for 92.7% ± 1.02% of FAME-derived lipids in the exponential phase Np1710/11 and 88.74% ± 0.6% in the stationary phase (Figure 3A,C). The wild-type strain by comparison only accumulated 7.34% ± 0.26% C17 in the exponential phase and 8.98% ± 1.1% in the stationary phase. Therefore, the results indicate alkane sequestration in LDs is enhanced by multiple copies of *aar/adc,* with a subsequent reduction in the proportion of C18:0 fatty acids in total FAMEs derived from neutral lipid extracts (Figure 2) that is due to their reduced proportions in LDs (Figure 3).

### 2.2. Phenotypic Effects of Aar/Adc Over-Expression

**Figure 4:**
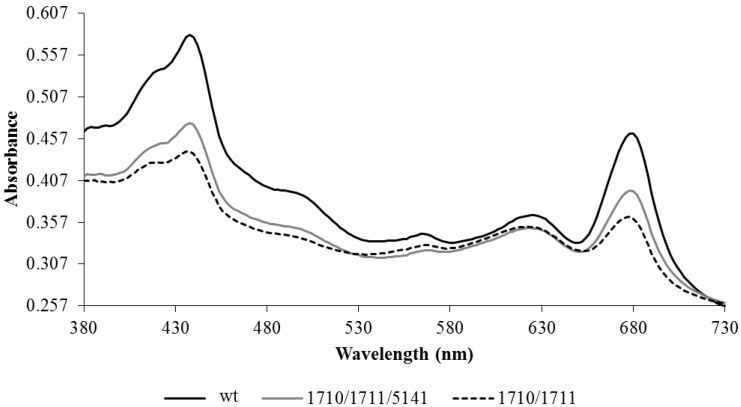
Representative whole cell absorption scans of wild-type (wt) *N. punctiforme* and strains bearing plasmid copies of Npun_F1710/11, alone or in combination with Npun_5141, under normal growth conditions in log phase.

The Np1711/10 strain had aberrant pigmentation and appeared bluish in color compared to the typical green color of the wild-type strain. To assess relative pigmentation differences, whole-cell spectral absorption scans were performed on cells normalized to cell density at 730 nm (Figure 4). All pigments were reduced in the strain-bearing Npun_R1710/11, but the losses were not equally distributed among the pigments. The light harvesting phycobilisome pigments phycocyanin and phycoerythrin showed only a small reduction in the alkane over-expressing strain relative to the wild-type strain. Chlorophyll and carotenoids were more severely reduced in the over-expressing strain relative to the wild-type strain.

**Figure 5:**
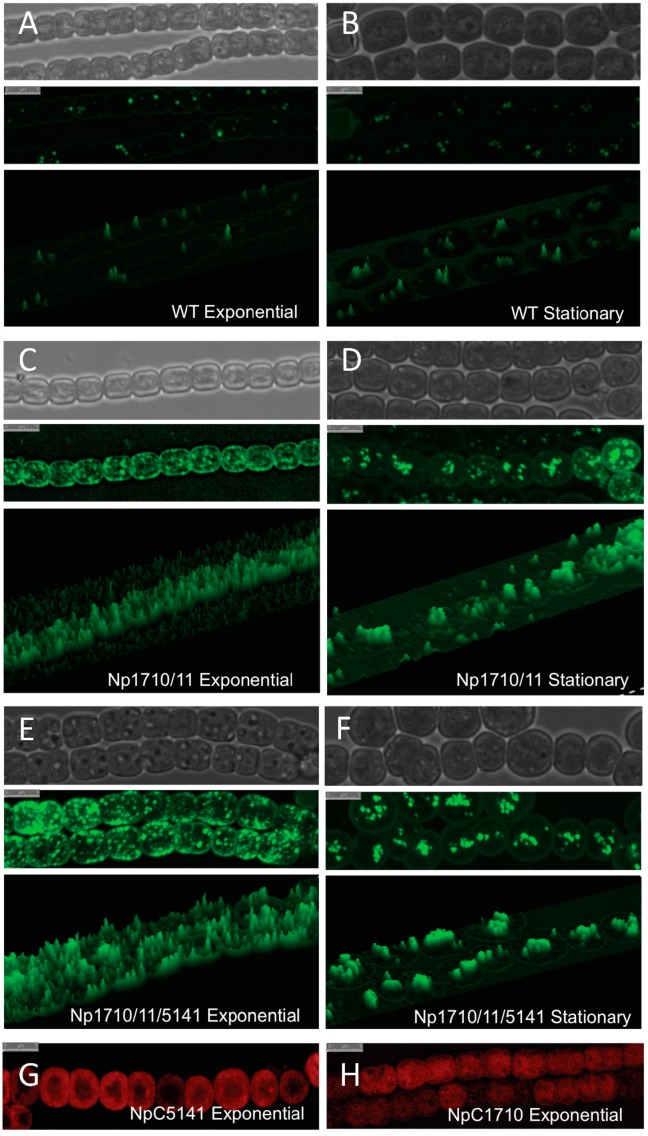
Confocal micrographs of Bodipy-stained lipid droplets and CFP-tagged proteins in *N. punctiforme*. Visible (top), Bodipy fluorescence (middle), and 3-D enhanced Bodipy fluorescence (bottom) micrographs of wild-type ((**A**) and (**B**)), Np1710/11 ((**C**) and (**D**)), and Np1710/11/5141 ((**E**) and (**F**)) strains during exponential (left panels) and stationary growth phase (right panels). CFP fluorescence of NpC5141 (**G**) and NpC1710 (**H**) during exponential growth phase. Scale bars denote 5 μm.

The Np1711/10 strain also exhibited a growth defect and aberrant LD formation. Under photoautotrophic growth conditions, Npun_F1710/11 grew 1.52-fold slower than the wild-type strain during log phase, doubling every 2.45 days compared 1.61 days for the wild-type strain. The Np1711/10 strain also expressed an abnormally large number of LDs, preferentially located near the periphery of cells during exponential growth (Figure 5C). During the stationary phase of growth, the numerous small LDs located around the periphery of the cell were fewer in number and were replaced by larger more fluorescent LDs that were centrally located (Figure 5D). This is in stark contrast to the wild-type strain that produces no visible peripheral LDs and fewer larger centrally located LDs regardless of the growth phase (Figure 5A,B).

### 2.3. Discovery and Use of a Novel Gene Enhancing Alkane Production

Since alkanes showed preferential deposition in cyanobacterial LDs, an attempt was made to identify LD-associated proteins contributing to alkane production. Proteins were found to be present in de-lipidized LDs observable by SDS-PAGE (data not shown). Mass analysis of an excised gel fragment identified Npun_R5141, a protein similar to a poorly characterized alpha-beta-hydrolase protein family containing hydrolases of diverse function [24], and containing two c21494 domains similar to esterases and lipases that act on carboxylic esters [25]. This 310 amino acid protein is one of 26 alpha-beta hydrolase proteins annotated in the genome of *N. punctiforme* and contains no predicted signal sequence or trans-membrane domains. All orthologs in other cyanobacteria contain annotated translational starts aligning to the methionine at position 13 of Npun_F5141, indicating this may be the actual *in vivo* start codon.

Lipids and alkanes extracted from whole cells were used to analyze the physiological effects of Npun_F5141 overexpression, both alone and in conjunction with Npun_R1710/11. Addition of extra copies of Npun_F5141 on a multi-copy plasmid resulted in wild-type relative expression levels of FAMEs and alkanes under normal light (Figure 2A), but a slight 1.35-fold increase in relative heptadecane production after four days of exposure to high light compared to a wild type control (Figure 2B). To see if over-expression of Npun_F5141 could enhance alkane production in conjunction with *aar/adc*, this gene and its promoter region was cloned into a multi-copy plasmid along with Npun_F1710/11 to create strain Np1710/11/5141.

Under normal light conditions, the exponentially growing Np1710/11 strain made 54.9% ± 0.73% heptadecane compared to other fatty acids in the cell (Figure 2A), whereas the Np1710/11/5141 strain made only 38.77% ± 2.3% heptadecane. This decrease in relative C17 production was due to an increase in saturated C16 and C18 fatty acids (Figure 2A). By removing the C17 data from analysis, it can be seen that addition of Npun_F5141 in conjunction with Npun_F1710/11 had the effect of increasing saturated C16:0 and C18:0 fatty acids in whole cells to above wild-type proportions under normal light while, at the same time, decreasing the relative amounts of unsaturated fatty acids (Figure 2C). Since the Np1710/11/5141 strain exhibited the highest NP alkane production of 6.9% ± 0.53% of cell dry weight (Figure 1), the lower *relative* C17 production must be due to increased fatty acid production and supports the hypothesized lipase function of Npun_F5141.

Under HL conditions, Np1710/11/5141 increased only 1.14-fold over Np1710/11 to relative production of 60.9% ± 0.33% (Figure 2B); however, in terms of overall production, the Np1710/11/5141 strain exhibited a total C17 content of 12.9% ± 0.30% dry weight. This equates to 11.5-fold increased production over wild-type levels under HL when the three genes are present in one strain, and a 16.8-fold increase over wild-type under NL (Figure 1).

Npun_F5141 has positive effects on the pigment and reduced exponential growth phenotype of the Np1710/11 strain. The Np1710/11/5141 strain was greenish-blue in color and had a less severe pigmentation defects as compared to Np1710/11 (Figure 4). In the Np1710/11/5141 strain carotenoids, phycoerethrin, and phycocyanin were similar to Np1710/11 and chlorophyll*a* was not reduced as severely as in Np1710/11. Npun_F5141 had positive effects on growth when added to the Npun_R1710/11 background, decreasing the doubling time from 2.45 days to 1.93 days under normal light. This was an increase in growth from 1.9-fold to only 1.2-fold slower than the wild-type strain.

The LD phenotype of Np1711/10/5141 strain was similar to that of Np1710/11 and expressed an even higher number of LDs preferentially located near the periphery of cells during exponential growth (Figure 5E). During stationary phase, these were replaced by larger more fluorescent LDs that were centrally located (Figure 5F).

### 2.4. Exogenously Supplied Carbon Enhances Growth but Not Alkane Production

Addition of exogenous fructose increased the growth rate of both wild type and over expresser strains. Under normal photoautotrophic growth conditions in the presence of exogenous fructose (mixotrophy), Np1710/11 increased its exponential growth rate, doubling every 1.7 days as compared to 2.45 days for photoautotrophy. The wild-type strain also increased growth with fructose, doubling every 1.4 days as compared to 1.61 days for photoautotrophy. Mixotrophy, therefore, allowed the Np1710/11 expression strain to grow at a rate only 1.2-fold slower than that of the wild type.

It was hypothesized that mixotrophic growth would also lead to enhanced alkane accumulation under exponential growth. However, when grown with additional carbon, wild-type cells in log phase accumulated only 0.40% ± 0.025% dry weight heptadecane in normal light and 0.71% ± 0.056% under high light conditions, while the Npun_R1710/11 over expresser strain accumulated 4.19% ± 0.5% heptadecane in normal light (Figure 1). Addition of fructose to the NpF1710/11/5141 strain yielded less heptadecane in log phase (5.21% ± 0.22%) compared to normal photoautotrophic growth conditions and an insignificant change (12.6% ± 0.91%) under high light conditions (Figure 1). The results, therefore, indicate that under normal light, mixotrophy could fix growth defects caused by excess copies of genes leading to alkane synthesis, however, alkane over-accumulation per dry weight was not enhanced. Unexplainably, high light, mixotrophic growth conditions caused the NpF1710/11 strain to die, however this could be abrogated if the Npun_ F5141 gene was also added (Figure 1).

### 2.5. Cellular Localization of Proteins Enhancing Alkane Production

To identify the cellular localization of proteins enhancing alkane production, the cerulean fluorescent protein (CFP) was fused to the C-terminal end of Npun_R1710 and Npun_F5141 on a multicopy plasmid under control of their own promoters and electroporated into the wild-type strain. Npun_R1710 was used as an indicator of Aar/Adc localization.

Confocal microscopy of Npun_R1710-CFP-tagged protein-reporter strain, C1710, indicated it is a soluble protein located throughout the cell (Figure 5H). It is expressed strongly during the exponential phase but CFP fluorescence declines during stationary phase to background levels (data not shown). The Npun_F5141-CFP-tagged protein-reporter strain, C5141, indicated that Npun_F541 localized to the periphery of the cell (Figure 5G).

## 3. Discussion

*N. punctiforme* has the ability to produce a large amount of a C17 alkane (Figure 1). *Synechocystis* sp. strain PCC6803 by comparison produces only 0.14% dry weight as alkanes, and addition of the Npun_F1710/11 genes increased this to 1.3% [23]. Here, we show *N. punctiforme* produces a higher starting amount of a single species of alkane under normal laboratory growth conditions, that could be increased 5.3-fold by extra copies of Npun_R1710/11, and nine-fold if Npun_F5141 was also added. High light could further increase total alkane production 1.5-, 2.1-, and 1.9-fold in wild-type, Np1710/11, and Np1710/11/5141 strains, respectively, for a maximal expression of 12.9% of cell dry weight in HL grown Np1710/11/5141 (Figure 1). Although supplementation with sugar could not further enhance total alkane production on a dry weight basis (Figure 1), increased growth rate by sugar addition would lead to increased total production for a given time period. Increased growth, but not LD formation by adding sugar, indicates growth is probably limited by resource drain of carbon into LDs in the alkane over-expressing cells and not by the toxic effects of alkanes. Cyanobacterial production systems have the capacity to produce alkane (Figure 1) and LDs during exponential growth (Figure 5), as well as the ability to fix their own nitrogen [26] unlike algal production systems that typically use nitrogen starvation to induce LD formation. Production platforms utilizing exponentially growing cells will be essential in the future to enhance synthesis of compounds requiring active metabolism for their synthesis.

This work extends previous work from our own lab that *N. punctiforme* produces a C17 alkane [14] and that of others showing multiple copies of the *aar/adc* enhances alkane production [22,23,27,28] by providing evidence for LD mediated alkane trafficking. During the exponential phase of growth a large number of small less fluorescent LDs are observed throughout the cell with enrichment at the cell periphery, whereas during stationary phase, these are replaced by multiple LDs that are centrally located and have a larger volume (compare Figure 5C,E with B,D). When overproduced, C17 is deposited almost exclusively in stationary phase LDs (Figure 3A), however during exponential phase, there is a large increase in the amount of alkanes that co-purifies with cell debris after LD removal (Figure 3C). These results support the hypothesis that smaller alkane-containing LDs are in the process of formation and are still attached to membrane systems within the cell. After alkane synthesis stops, they make their way to existing LDs or mature into large non-membrane associated LDs. This is supported by the abundance of CFP-labeled Npun-F1710 that parallels that of the small LDs, showing maximal expression during the exponential phase and minimal expression during the stationary phase.

We attribute the increased overall production of alkanes in *N. punctiforme* to a combination of the following contributing factors: (1) A higher initial starting alkane concentration, and an assumed underlying metabolism supplying substrates for Aar/Adc; (2) A higher gene copy number, since the parent plasmid pSCR119 has been determined to be 12-14 copies per chromosome (Summers) as opposed to only two chromosomally located copies of the *aar/adc* genes in the *Synechocystis* study; (3) The use of high light to boost photosynthesis, and CO_2_-derived fatty acid production; (4) A high specific enzymatic activity of Aar/Adc from *N. punctiforme,* the highest observed among orthologous proteins from eight cyanobacterial strains [22]; (5) sequestration of potentially toxic levels of alkanes into LDs; and (6) The discovery and use of an additional novel gene showing homology to protein families possessing esterase/lipase activities. We expect further improvements to alkane production could be achieved by additional metabolic alterations leading to over production of fatty acyl-ACP substrate for Aar/Adc by *de novo* synthesis [27] and/or enhanced recycling of free fatty acids [23].

CFP-labeled Npun_F1710 was observed throughout the cell during exponential growth (Figure 5H), indicating it is a soluble enzyme or potential interaction/association of this alkane biosynthetic protein with the thylakoid membrane system. It is likely that over-production of small LDs and increased amounts of large LDs occur as a protective mechanism in response to increased alkane production as a way to sequester hydrocarbons that might otherwise destabilize functional membrane systems. The high concentration of nascent LDs at the cell periphery indicates the cell membrane may be the site of LD biogenesis, however detailed electron microscopy will be required to observe LD budding and determine if the cell membrane is truly the origin of LDs as this data suggests.

Pigmentation defects due to over-production of C17 are a result of reduced chlorophyll*a* and carotenoid levels in proportion to phycobilisomes (Figure 4). Since the fatty acid profiles from membranes remaining in the pellet do not differ as a result of alkane overproduction (Figure 3B,D), it is more likely that the small amount of alkanes present in membranes found in the 1710/11 pellet (Figure 3A) causes this effect rather than lipid imbalance. Long chain alkanes insert in the membrane parallel to the fatty-acyl side chains of lipids and increase the transition temperature, thus, decreasing fluidity [29]. Photosystems require interactions with lipids with the correct fluidity [30], for example the requirement of unsaturated lipids for proper insertion of the D1 protein into the PS II complex after light damage [31]. The drastic increase in lipid desaturation, which occurred in all strains due to high light (Figure 2C,D) could act to abrogate the potentially harmful effect of alkanes in photosynthetic membranes and allow even higher alkane production.

Sole-production of C17, even when overexpressed to over 12% of cell dry weight, implies additional species-specific components may interact with Aar/Adc in *N. punctiforme*. Sole production of C17 is unique in light of reports that these same two genes when expressed in other organisms exhibited broad substrate specificity. For instance when Npun_R1710 and Npun_R1711 were expressed in *E. coli*, a mixture of C13 and C15 alkanes and C15 and C17 alkenes were produced [22], and when supplemented with a branched-chain fatty acid, Npun_R1710 and Npun_R1711 were capable of producing branched chain alkanes [28]. When these same two *N. punctiforme* genes were expressed in the unicellular cyanobacterium *Synechocystis* sp. strain 6803, both C17 alkanes and alkenes were produced [23]. Interactions of NpF1710/11 with protein components unique to this strain that preferentially provide the C18:0-ACP substrate could explain these results, however the exact mechanism remains to be elucidated. The data suggests a working model proposing that the Aar/Adc enzymes coopt newly synthesized 18-carbon long fatty acids destined for deposition in LDs. When *aar/adc* are overproduced, C18:0 fatty acids are depleted specifically from LDs, and do not alter the composition of pellet membranes (Figure 3B,D) indicating the site of synthesis and conversion to alkanes is likely near LDs. Since lipids are synthesized in their saturated form and double bonds are introduced by desaturases after synthesis, this co-localization model would explain the sole synthesis of C17 from fatty acids destined for the LD. The alternate hypothesis that alkanes arise by the recycling of membrane lipids cannot be discounted, but would require re-saturation of unsaturated fatty acids released from membranes followed by specific use of only C18:0. There is no evidence for specific lipases releasing 18:0-only fatty acids since pellet lipids are un-changed in the Np1710/11 strain (Figure 3B,D).

The mechanism explaining enhanced alkane production of Npun_F5141 only when in conjunction with Npun_R1710/11 (Figure 2) remains to be elucidated, however its postulated role as a lipase involved in lipid recycling would explain this synergism. The lipid recycling enzyme acyl-ACP synthetase (Acs) normally activates free fatty acids for lipid biosynthesis by attaching them to the acyl carrier protein [32]. Increased free fatty acids produced by a lipase in the Np5141 strain would be expected to increase the pool of acyl-ACP for recycling back into membrane lipids, thus, explaining why it had little effect on alkane production in the Np5141 strain (Figure 2A,B). However, in Np1710/11/5141 this pool could be utilized by Aar/Adc for increased alkane production (Figure 2A,C). Previous work showing enhanced alkane production through acyl-ACP pool enhancement by Acs over-production supports this line of reasoning [23]. Localization of Npun_F5141 near the cell membrane supports its involvement in membrane lipid recycling. Efforts are underway to determine the enzymatic function of Npun_F5141 and its effect on the composition of LDs when expressed with Npun_R1710/11.

## 4. Materials and Methods

### 4.1. Strains and Growth Conditions

*Nostoc punctiforme* PCC 73102 cultures were grown in 125 mL or 50 mL Erlenmeyer flasks shaking at 100–120 rpm at 25 °C under 13–15 µmol s^−1^ m^−2^ (normal light; NL) or 135–160 µmol s^−1^ m^−2^ (high light; HL) from cool white fluorescent lights using the medium of Allan and Arnon [33], diluted four-fold. Flasks were supplemented with one of the following additions (pH 7.8); 5 mM MOPS 2.5 mM Ammonia (MA) or 5 mM MOPS 2.5 mM Ammonia /20 mM Fructose (MAF). Exponential growth rates were established from triplicate flasks using growth curves measuring chlorophyll*a* concentrations of cultures over 8 days.

For Heptadecane accumulation determination, triplicate cultures were grown in MA or MAF shaking at 100 rpm in 50 mL Erlenmeyer flasks and sampled during log phase (3.8–5.1 µg Chl*a*/mL for wild type 1.6–2.6 µg Chl*a*/mL for heptadecane over expresser strains) or stationary phase (23–30 µg Chl*a*/mL for wild type 12–14 µg Chl*a*/mL for heptadecane over expresser strains). Chlorophyll*a* readings corresponding to log and stationary phase were normalized using growth curves created for both over expresser and the wild type strains.

### 4.2. Plasmid and Strain Construction

Genes of interest under control of their own promoters were amplified from the *N. punctiforme* genome and over expressed by cloning into pSCR119, a shuttle plasmid capable of replication in both *Escherichia coli* and *Nostoc punctiforme.* All inserts were PCR amplified using Herculase polymerase (Agilent Technologies), cloned using standard techniques [34], and were verified by sequencing. Plasmids were introduced into *N. punctiforme* by electroporation as previously described [35]. The Npun_F1710/11 expression strain was made by PCR amplifying adjacent genes Npun_R1711 and Npun_R1710 using primers (P1-ATACTGCAGACCGCAAAGCATTTATTTCG and P2-TTTGGTACCTTGTCCTTTGTCCTTTGTCC) and subsequent cloning into the *PstI* / *KpnI* sites of pSCR119 [35] to create plasmid pNp1710/11. To create the Npun_F5141 expression strain, Np5141 was PCR amplified using primers P1- CTGCTGCAGCATGACATCCCGCCTTACCA /P2-AATGGTACCTGGTAATAGACAGGGCTAGGACT and cloned into the sites of pSCR119. The resulting plasmid, pNp5141, was electroporated into *N. punctiforme*. To created strain Np1711/10/5141, Npun_F5141 was amplified with primer P1-AGCGCATGCCATGACATCCCGCCTTACCA and P2-AGCCTGCAGTGGTAATAGACAGGGCTAGGACT, cloned into the *SphI*/*PstI* sites of pNp1710/11 and electroporated into *N. punctiforme.* The empty reporter plasmid pSUN-CERC was made by PCR-amplifying the upstream terminator, multiple cloning sites, and cerulean fluorescent protein (CFP) reporter gene from pCERC-1 [36] with primer pair P1-CTTGCATGCCAAATAAAACGAAAGGCTCAGTCGAA/P2-TTCCCCGGGTTACTTGTACAGCTCGTCCATGCC. The multiple cloning region of pSCR119 was removed by using restriction enzymes *SphI* and *Ecl136II* and replaced with the PCR product digested with *SphI* and *SmaI*. To create the C5141 reporter strain, the Npun_F5141 gene was amplified without its stop codon with primer set P1-CTGCTGCAGCATGACATCCCGCCTTACCA/P2-ACCGAGCTCTAAGCAAAGGTAAAAATTGG) and cloned in frame with the downstream CFP protein on pSUN-CERC using restriction enzymes *PstI* and *SacI*. The resulting plasmid pC5141 was electroported into *N. punctiforme* to make strain C5141. To create the C1710 reporter strain, the Npun_R1710 genes were amplified with primer set (P1-ATACTGCAGACCGCAAAGCATTTATTTCG/P2-ATGGAGCTCCCAACAACGGTCTAAATCCAT) and cloned in frame with CFP using restriction enzymes *PstI* and *SacI.* The resulting plasmid pC1710 was electroporated into *N. punctiforme* to make strain C1710.

### 4.3. Microscopy

Lipid droplets are not obvious using brightfield or phase contrast microscopy and so were stained using the fluorescent dye borondipyrromethene difluoride (BODIPY) 505/515 (Invitrogen Molecular Probes, Carlasbad, CA, USA) specific for neutral lipids and visualized by confocal microscopy as previously described [14]. Cerulean tagged proteins were visualized using a 458 nm laser excitation line and a window of 468–488 nm for visualization. Maximum projection of Z-stacks was used to produce images. Co-localization data was collected by first visualizing for CFP fluorescence followed by BODIPY fluorescence.

### 4.4. Relative Heptadecane and Fatty Acids Composition of Whole Cells, LDs, and Cell Pellets

Whole cells containing 100–200 µg Chl*a* were harvested by centrifuging at 6000× *g* for five minutes, washed twice with deionized water, and concentrated to 0.75 mL in a 2 mL conical screw cap tube. Cells were extracted with 1 mL of 75% iso-octane/25% ethyl acetate (v/v) using a bead beater for 100 s at maximum power with 0.5 g of 0.5 mm glass beads. Lysed cells were centrifuged for 2 min at 13,000× *g* and 0.75 mL of the top layer containing heptadecane and neutral lipids were removed, dried under nitrogen gas, and converted to fatty acid methyl esters (FAMEs) by saponification with 1 mL of 1 N KOH in anhydrous methanol at 80 °C for 30 min. Methyl esterization was accomplished by adding 1 mL of BCl_3_-Methanol (12% W/W) and heating 100 °C for 10 min. After cooling, 10 µL of 1 M HCl, 1 mL water and 1 mL hexane was added and vortexed violently for 1 min to extract fatty acid methyl esters FAMEs into the non-polar solvent. The upper organic layer was removed and dried under a stream of N_2_ gas [37]. FAMEs were re-dissolved in hexane and analyzed by GC-MS using a Restek Rxi-5Sil MS column (0.25 mm ID × 30 m, 0.10 µm film thickness). Identifications were confirmed using NIST11/NIST11s libraries and commercial FAME standards (RESTEK/ Cat#35066).

For LD composition analysis LDs from cells containing 200–400 µg Chl*a* were harvested using centrifugation and analyzed according to a previously published method [14]. Heptadecane and FAMEs were analyzed by GC-MS using a Restek RX1-5Sil MS column (0.25 mm id × 30 m, 0.10 µm film thickness) and confirmed as above.

### 4.5. Heptadecane Quantitation

Heptadecane production in wild type control and over expresser strains exponentially growing in MA were used for analysis. Cultures were aliquoted into separate flask and one set was grown in the presence of 20 mM fructose for two days while the other set grew without any added carbon. For HL cultures experiments in MA and MA/fructose cultures (10 mL of cells in 50 mL flask) were placed under high light for four days while the other set grew under normal light. After each experiment, 4 mL of cells were washed twice with deionized water and pelleted for lipid extraction while 2 mL of cells were washed twice, pelleted, dried in a vacuumed oven at 80 °C for 1.5 h, and weighed. Cell pellets were extracted as above after the addition of 12 µL of eicosane internal standard (1.5 mg/mL in hexane) and analyzed by GC-MS. The amount of heptadecane presented in each sample was determined by comparison to a heptadecane standard curve. Extraction efficiencies were determined using an eicosane standard curve.

### 4.6. Whole-Cell Spectral Analysis

To discern alterations in pigments due to alkane over-production exponentially grown whole-cells in media were subjected to absorption scans from 380 to 730 nm using a Beckman Coulter DU 640 spectrophotometer. Raw absorption values from the scan data were used to crudely estimate alterations in various pigments between strains using 480 nm for beta-carotene [38], 564 nm for phycoerythrin, 620 nm for phycocyanin, and 678 nm for chlorophyll*a* [39]. All cultures were normalized to an absorption of ~0.26 at 730 nm prior to wavelength scanning.
